# Hypothalamic activation is essential for endotoxemia-induced acute muscle wasting

**DOI:** 10.1038/srep38544

**Published:** 2016-12-06

**Authors:** Kaipeng Duan, Qiyi Chen, Minhua Cheng, Chenyan Zhao, Zhiliang Lin, Shanjun Tan, Fengchan Xi, Tao Gao, Jialiang Shi, Juanhong Shen, Weiqin Li, Wenkui Yu, Jieshou Li, Ning Li

**Affiliations:** 1 Department of General Surgery, Jinling hospital, Medical School of Nanjing University, Nanjing, 210002, P.R. China

## Abstract

Growing evidence suggests acute skeletal muscle wasting is a key factor affecting nutritional support and prognosis in critical patients. Previously, plenty of studies of muscle wasting focused on the peripheral pathway, little was known about the central role. We tested the hypothesis whether central inflammatory pathway and neuropeptides were involved in the process. In lipopolysaccharide (LPS) treated rats, hypothalamic NF-κB pathway and inflammation were highly activated, which was accompanied with severe muscle wasting. Central inhibition of nuclear factor kappa-B (NF-κB) pathway activation by infusion of an inhibitor (PS1145) can efficiently reduce muscle wasting as well as attenuate hypothalamic neuropeptides alteration. Furthermore, knockdown the expression of anorexigenic neuropeptide proopiomelanocortin (POMC) expression with a lentiviral vector containing shRNA can significantly alleviate LPS-induced muscle wasting, whereas hypothalamic inflammation or NF-κB pathway was barely affected. Taken together, these results suggest activation of hypothalamic POMC is pivotal for acute muscle wasting caused by endotoxemia. Neuropeptide POMC expression may have mediated the contribution of hypothalamic inflammation to peripheral muscle wasting. Pharmaceuticals with the ability of inhibiting hypothalamic NF-κB pathway or POMC activation may have a therapeutic potential for acute muscle wasting and nutritional therapy in septic patients.

In critical patients, sepsis is prevalent and may cause severe complications, thus affecting the prognosis and quality of life. Sepsis is always accompanied by metabolic disorders, presenting a hypermetabolic state. Sustained hypercatabolism, reduced feeding and subsequent muscle atrophy will deplete body fat and protein reserves, leading to cachexia, impaired host immunity and increased mortality^1^. Nutrition support is employed to counteract the detrimental process and enhance the capability of body recovery. However, recently, two large clinical trials, EPaNIC and EDEN trial, yield poor results from different nutrition strategies, showing little effects of early parental feeding or full enteral feeding in critical patients^2^,^3^. These results questioned the type, quantity and timing of nutrition support in critical illness. Actually, multiple factors can affect metabolic and nutritional conditions, including the loss of *ad libitum* feeding, intolerance of nutrition support and hypercatabolism. Lately, emerging translational data have indicated the negative nutritional results are highly related to the hypercatabolism, particularly acute muscle wasting, in these patients^4^. Acute skeletal muscle wasting occurred early in critical patients regardless of the timing of feeding strategy^5^,^6^. Also, recent studies^7^,^8^ have shown muscle wasting in critical patients is closely associated with the prognosis. Therefore, acute skeletal muscle wasting is an important component of metabolic and nutritional issue in critical patients. And elucidation of the mechanism underlying the muscle wasting is necessary for the amelioration of metabolic disorder and the improvement of life quality in these patients.

Although previous researches have suggested peripheral inflammation participated in sepsis-induced weight loss^9^, few studies have examined the role of central nervous system (CNS) in the process as it has been in context of obesity and type 2 diabetes^10^,^11^. Since both appetite and body weight are regulated by the CNS, especially the arcuate nucleus (ARC) of hypothalamus^12^, the weight loss in sepsis may also be mediated via a central mechanism. In concert with this hypothesis, a study have demonstrated central melanocortin system exerted a critical role in the regulation of body weight and cachexia induced by tumor^13^. In addition, it has been documented that central inflammation is both essential and sufficient to induce muscle atrophy^14^,^15^. However, at present, the exact central mechanism triggering acute muscle wasting during infection remains unknown.

Melanocortin system contains two important neurons, POMC and agouti-related protein (AgRP)^16^. The peptide precursor POMC can be further cleaved into α-melanocyte-stimulating hormone (α-MSH), which agitates central melanocortin receptors, including type-4 melanocortin receptor (MC4R) and MC3R, to increase energy expenditure and reduce body weight. AgRP acts as an antagonist of MC4R, and central administration of AgRP could ameliorate muscle wasting and systemic inflammation caused by chronic kidney disease^17^. Moreover, both the neurons are subjected to proinflammatory cytokines and circulating molecules, such as insulin and leptin, to regulate energy balances^10^,^12^.

IKKβ/NF-κB is a pivotal regulator of the expression of genes related inflammation and innate immunity^18^. In the resting state, NF-κB dimers exist in an inactive form in the cytoplasm bound to the IκBα inhibitory protein. Inflammatory stimuli activate the IκB kinase(IKK)complex, which phosphorylates IκB, leading to its degradation. This will facilitates translocation of NF-κB to the nucleus, thus regulating the transcription of genes. Plenty of studies have shown activation of IKKβ/NF-κB pathway in the hypothalamus is essential for the development of type 2 diabetes and obesity^11^. On the other hand, hypothalamic NF-κB activation is critical for the anorexia and neuropeptide gene expression caused by infection and tumor^19^. Since the reduced appetite and acute wasting were presented by critical patients simultaneously, we hypothesized that hypothalamic NF-κB pathway and neuropeptides were involved in the regulation of acute skeletal muscle wasting induced by sepsis, and tested whether inhibiting hypothalamic NF-κB activation or knocking down certain neuropeptide can mitigate the detrimental process.

## Results

### The effect of inhibiting central NF-κB pathway on hypothalamic inflammation expression

**Figure 1:**
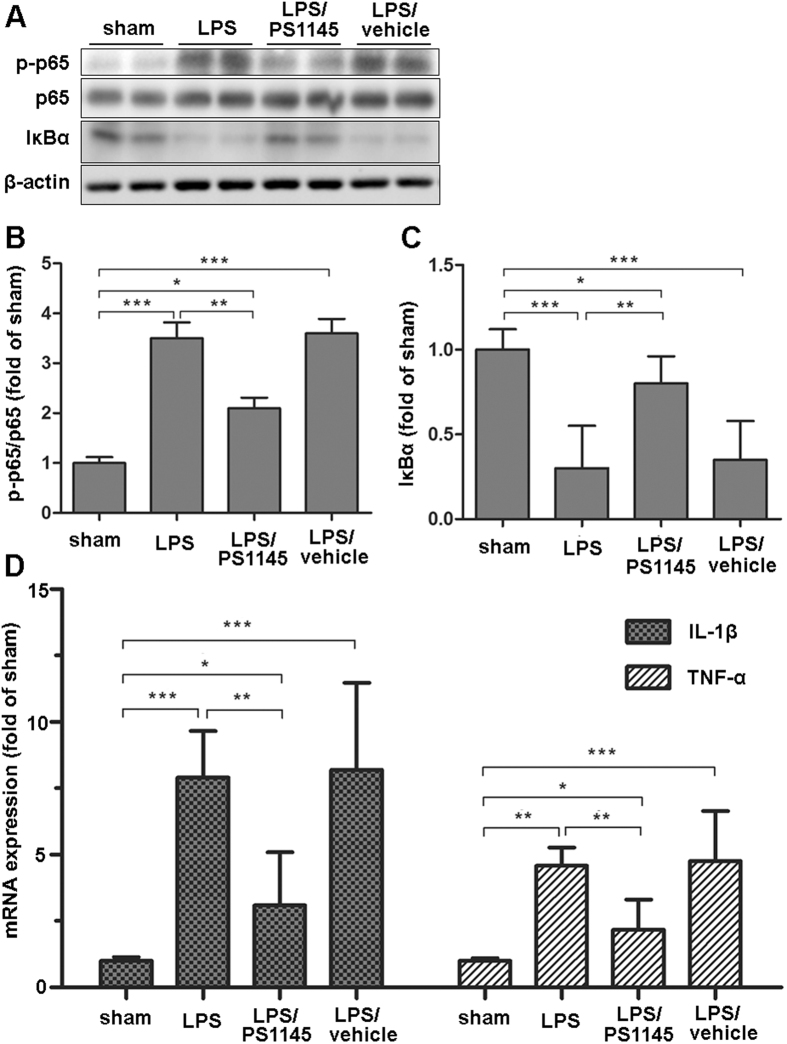
Effects of icv IKKβ inhibitor treatment on hypothalamic signaling pathway and inflammatory cytokine expression. Third ventricle-cannulated rats (n = 6–8/group) in experiment 1 were euthanized 24 h after the final icv injection for collection of hypothalamic tissue. Hypothalamic signaling pathway proteins (p-p65, p65 and IκBα) were measured by western blotting (**A** shown in cropped gels), and were normalized by the total protein levels of p65 or β-actin (**B** and **C**). Hypothalamic inflammatory cytokine (IL-1β and TNF-α) expression was measured by real-time PCR (**D**). Reported values are relative to GAPDH. Data are represented as mean ± SEM. *P < 0.05; **P < 0.01; ***P < 0.001.

To examine the effect of central IKKβ/NF-κB pathway on endotoxemia-induced muscle wasting, IKKβ inhibitor or vehicle was directly injected into the 3V of septic rats through a previously implanted cannula. Twenty four hours after administration, hypothalamic tissue was harvested for protein and mRNA measurement. As shown in Fig. 1, in the LPS group, phosphorylation levels of NF-κB subunit p65 significantly increased and the IκBα level decreased at 24 h after LPS injection when compared with the sham (Fig. 1A,B and C). Given the subsequent degradation of IκBα, these results indicated the activation of IKKβ/NF-κB pathway. As expected, in the LPS/PS1145 group, the level of phosphorylated p65 was reduced and total IκBα was higher than those of LPS and LPS/vehicle group (Fig. 1A,B and C). Since the close relationship between NF-κB pathway and inflammation^18^, we also examined the hypothalamic inflammation. LPS injection caused a marked elevation of IL-1β and TNF-α expression, while in the LPS/PS1145 group, hypothalamic inflammation was significantly reduced when compared with LPS/vehicle and LPS group (Fig. 1D). Collectively, these results demonstrated peripheral LPS injection could lead to hypothalamic IKKβ/NF-κB pathway activation and increased inflammation. Blockade of NF-κB pathway can attenuate hypothalamic inflammation.

### Blockade of central NF-κB pathway ameliorating peripheral muscle wasting induced by LPS administration

**Figure 2:**
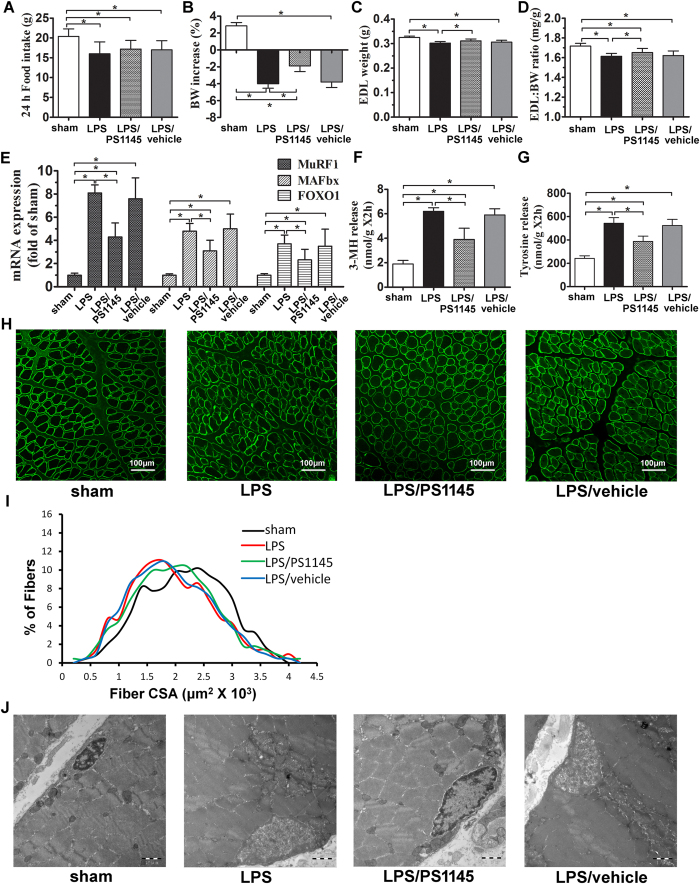
Inhibiting central IKKβ/NF -κB pathway can alleviate endotoxemia-induced muscle wasting. Twenty-four hours after treatment in experiment 1, rats were euthanized and EDL and gastrocnemius were harvested for measurement. Food intake (**A**), BW increase (**B**), EDL weight (**C**) and EDL weight normalized to initial BW (**D**) were measured. Atrophy gene (MuRF1, MAFbx and FOXO1) expression in rat gastrocnemius muscle were measured by real-time PCR (**E**). Reported values are relative to GAPDH. Net 3-MH and tyrosine release of EDL reflecting total protein breakdown were determined by HPLC (**F** and **G**). Gastrocnemius were immunostained for laminin (green) to delineate fiber area (**H**) and frequency distribution was calculated (**I**). Ultrastructure of myocyte and mitochondria under TEM (magnification*20000) (**J**). Data are represented as mean ± SEM. *P < 0.05; **P < 0.01; ***P < 0.001.

Previous studies have shown a critical role of hypothalamic inflammation on muscle atrophy^14^. Here, we tested the effect of central IKKβ/NF-κB pathway in the process. LPS caused a significant reduction of body weight (BW) and EDL-BW ratio (Fig. 2A–D). Meanwhile, genes related muscle atrophy, muscle ring finger 1 (MuRF1), muscle atrophy F-box (MAFbx) and forkhead box protein O1 (FOXO1), were significantly elevated at 24 hour after LPS administration (Fig. 2E), indicating severe muscle wasting. Also markers for protein turnover, 3-MH and tyrosine release, rose substantially in the LPS group (Fig. 2F and G). To examine whether the loss of muscle mass is the result of myofibrillar atrophy, muscle fiber cross section area (CSA) was measured in regions of the gastrocnemius (Gn) muscle composed of fast fibers. As demonstrated in Fig. 2H and I, CSA showed a pronounced reduction after LPS injection. Importantly, in the LPS/PS1145 group, rats showed a less reduction of body weight and EDL-BW ratio. Atrophic genes and markers for protein turnover were also attenuated when compared with LPS and LPS/vehicle group. Consistently, blockade of central NF-κB pathway showed a less reduction of CSA in Gn than LPS group (Fig. 2A–I). Under ultrastructure examination, LPS administration caused myocyte injury with dissolved myofilament, swollen nucleus and broken inner membrane cristae in mitochondria when compared with sham. However, those subcellular impairment was largely attenuated in rats treated with NF-κB pathway inhibitor (Fig. 2J). Taken together, these data suggested endotoxemia-induced muscle wasting was ameliorated by central NF-κB pathway inhibition. Meanwhile, we measured the plasma IL-6 and corticosterone of these subjects. As shown in Table 1, IL-6 increased significantly in all the LPS-injected rats. Corticosterone showed a similar pattern, but group LPS/PS1145 and group LPS differed statistically.

### The effect of inhibition NF-κB pathway on the expression of hypothalamic neuropeptides

**Figure 3:**
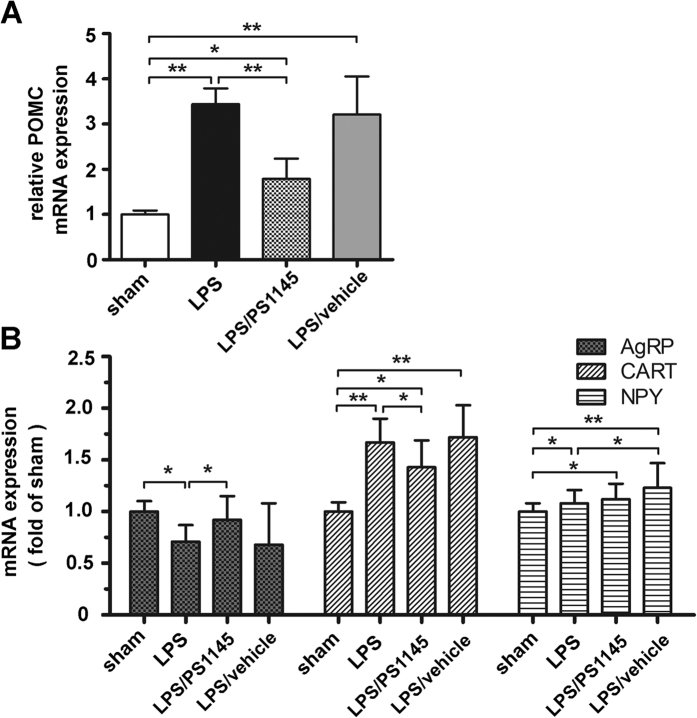
Effects of inhibiting central IKKβ/NF-κB pathway on hypothalamic neuropeptides changes. Real-time PCR analysis of hypothalamic POMC (**A**), AgRP, CART and NPY (**B**) gene expression was performed. GAPDH was used as control. Data are represented as mean ± SEM. *P < 0.05; **P < 0.01; ***P < 0.001.

Since hypothalamic melanocortin system are subjected to inflammation^12^, we also measured the expression of neuropeptides of melanocortin system. As shown in Fig. 3, LPS administration led to 3–4 fold increase of POMC expression, and reduced AgRP expression. Anorexigenic peptide cocaine- and amphetamine-regulated transcript (CART) and orexigenic peptide neuropeptide Y (NPY) also show higher expression than the control. However, in rats treated with LPS and NF-κB inhibitor, the elevated POMC expression was significantly lower than that of the LPS group, despite higher than the control. Similarly, the alteration of AgRP and CART expression caused by LPS injection were also substantially attenuated, even comparable to the sham. However, it seems that inhibition NF-κB has little effect on the NPY expression. Therefore, blockade of central NF-κB can affected several certain neuropeptides, in particular POMC and AgRP.

### Knockdown of hypothalamic POMC expression by a lentiviral method

**Figure 4:**
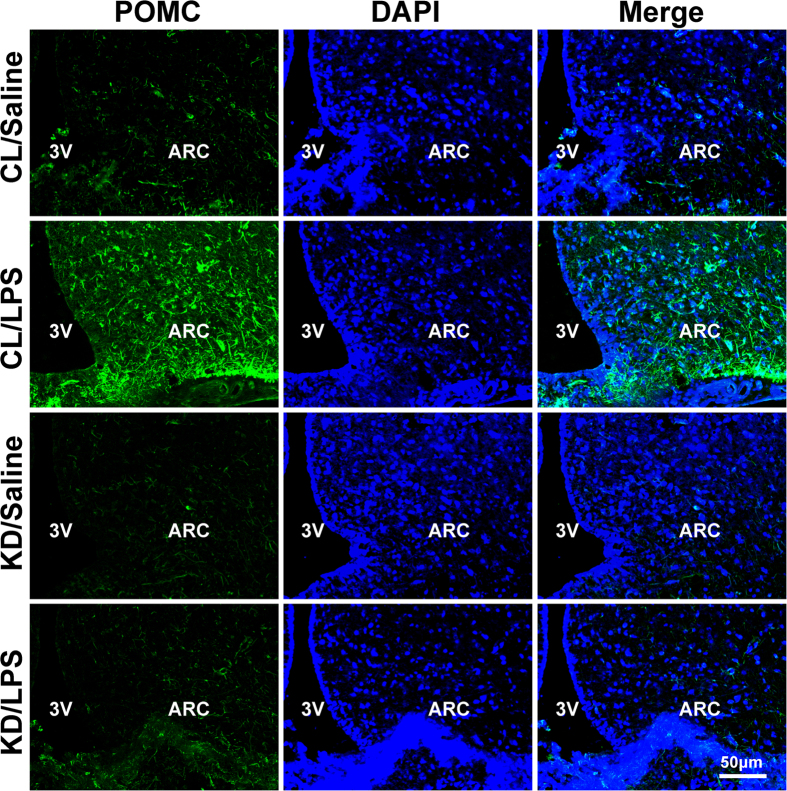
Hypothalamic neuropeptide POMC expression in saline or LPS treated rats after ARC injection of lentiviruses containing shRNA against POMC or control shRNA. Two weeks after ARC viruses injection, rats were i.p. injected saline or LPS. Twenty four hours later, brain tissue were harvested for immunostaining as described in method. Data show POMC immunostaining (green) across the hypothalamic ARC of rats in experiment 2. DAPI staining (blue) reveals the nucleus of all cells in the sections (**A**). Bar = 50 μm. A subset of rats were used for the detection of POMC mRNA expression by real-time PCR method (**B**). GAPDH was used as control. Data are represented as mean ± SEM. *P < 0.05; **P < 0.01; ***P < 0.001.

**Figure 5:**
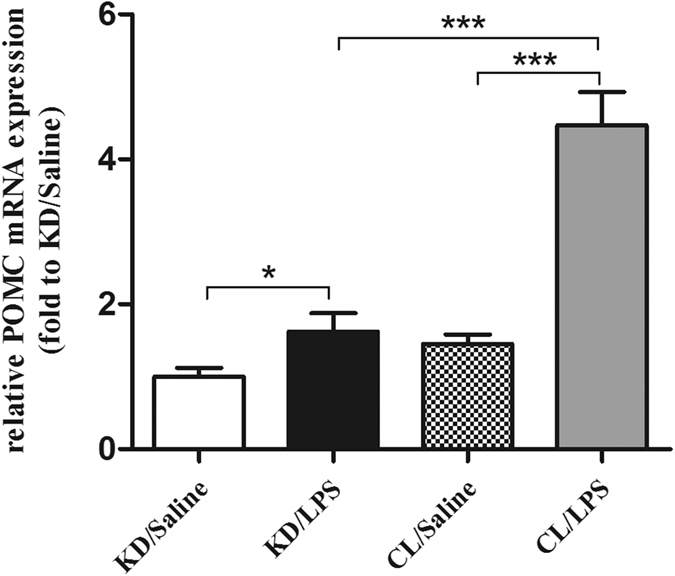
The expression of POMC mRNA in rats treated with saline or LPS after ARC injection of lentiviruses containing shRNA against POMC or control shRNA. Real-time PCR analysis of hypothalamic POMC gene expression was performed. GAPDH was used as control. Data are represented as mean ± SEM. *P < 0.05; **P < 0.01; ***P < 0.001.

Since inhibiting central NF-κB pathway can affect hypothalamic neuropeptides and alleviate peripheral muscle wasting simultaneously, we next questioned whether neuropeptides mediated the effect of central inflammation on peripheral atrophy. Due to the critical role of POMC in energy balance^20^ and good association in endotoxemia-induced muscle wasting as shown above, we employed a site-specific RNA interference to knock down POMC expression via a hypothalamic delivery of lentiviral shRNA against rat POMC. Normal rats were bilaterally injected with lentiviral POMC shRNA or matched control shRNA into the ARC. The rats received POMC shRNA or control shRNA were termed POMC KD (KD) or POMC CL (CL) rats, respectively. Using POMC immunostaining and mRNA test, we confirmed that site specific POMC knockdown was successful in POMC KD rats (Fig. 4 and Fig. 5).

### Knockdown of POMC effectively ameliorating peripheral muscle wasting induced by LPS

**Figure 6:**
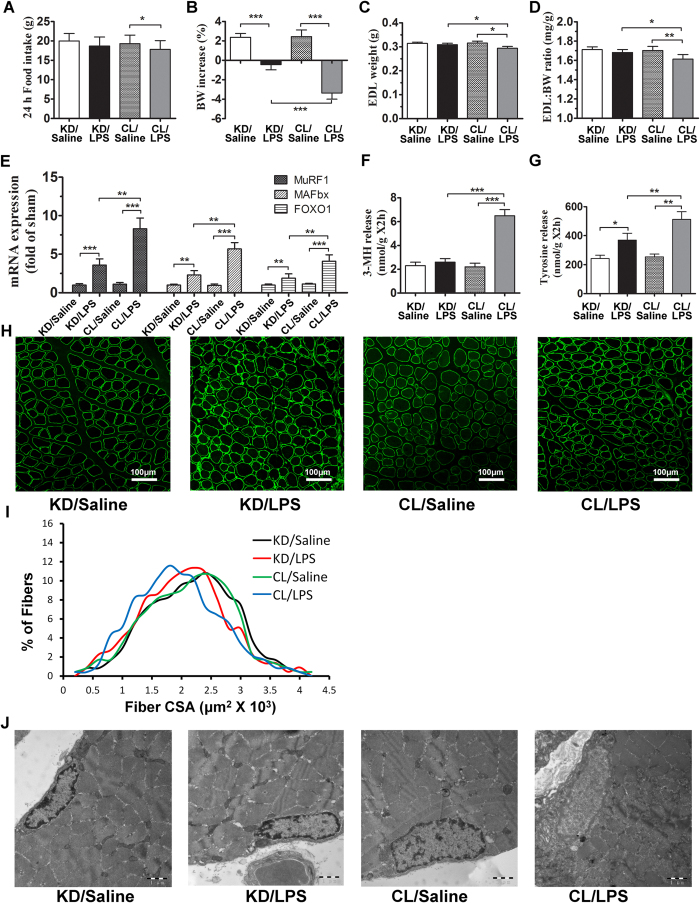
Knocking down hypothalamic POMC expression can effectively attenuate endotoxemia-induced muscle wasting. Twenty-four hours after treatment in experiment 2, rats were euthanized and EDL and gastrocnemius were harvested for measurement. Food intake (**A**), BW increase (**B**), EDL weight (**C**) and EDL weight normalized to initial BW (**D**) were measured. Atrophy gene (MuRF1, MAFbx and FOXO1) expression in rat gastrocnemius muscle were measured by real-time PCR (**E**). Reported values are relative to GAPDH. Net 3-MH and tyrosine release of EDL reflecting total protein breakdown were determined by HPLC (**F** and **G**). Gastrocnemius were immunostained for laminin (green) to delineate fiber area (**H**) and frequency distribution was calculated (**I**). Ultrastructure of myocyte and mitochondria under TEM (magnification*20000) (**J**). Data are represented as mean ± SEM. *P < 0.05; **P < 0.01; ***P < 0.001.

As shown in Fig. 6, after LPS administration, POMC KD rats showed significantly less decrease of EDL-BW ratio, EDL weight as well as body weight reduction when compared with POMC CL rats (Fig. 6A–D). Concurrent with the alteration of body weight, the expression of atrophic genes, MuRF1, MAFbx and FOXO1, were also much lower in POMC KD rats (Fig. 6E). Similarly, protein turnover was largely attenuated, as lower 3MH and tyrosine level were detected in POMC KD rats (Fig. 6F and G). In CSA measurement, the fiber size of POMC KD rats were also preserved when compared with the control (Fig. 6H and I). However, in rats received saline injection, there was no significant difference in body weight, atrophic genes expression or protein breakdown between POMC KD and POMC CL rats (Fig. 6A–I), suggesting knockdown POMC expression have little impact on muscle wasting in non-septic rats. Similarly, under TEM examination, the subcellular injury, such as dissolved myofilament, swollen nucleus and decreased mitochondria, presented in POMC CL rats were much ameliorated in POMC KD rat after LPS challenge (Fig. 6J). As before, LPS treatment increased plasma IL-6 level significantly. Noticeably, corticosterone in KD/LPS group was much lower than that of CL/LPS group (Table 2).

### Hypothalamic NF-κB pathway and inflammation caused by LPS were unaffected by the Knockdown of hypothalamic POMC

**Figure 7:**
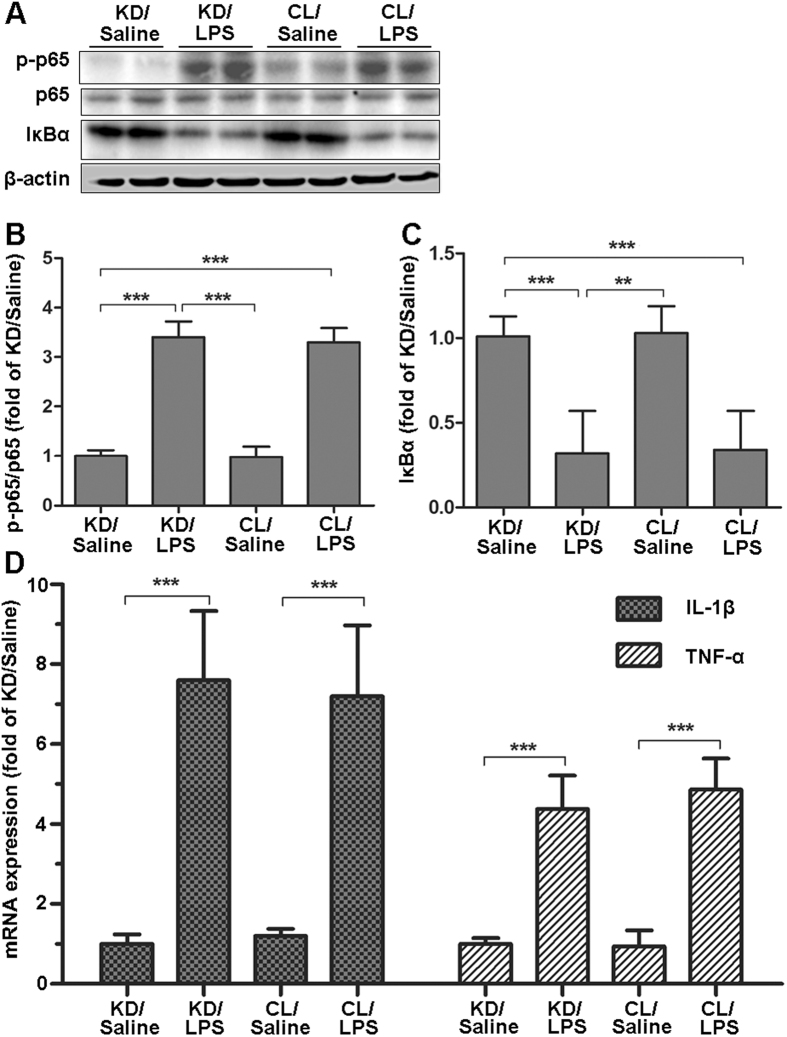
Knocking down hypothalamic POMC expression does not affect central NF-κB signaling pathway and inflammatory cytokine gene expression caused by LPS or saline. Twenty-four hours after treatment in experiment 2, rats were euthanized and hypothalamus were collected for measurement. Hypothalamic signaling pathway proteins (p-p65, p65 and IκBα) were measured by western blotting (**A**, shown in cropped gels) and were normalized by the total protein levels of p65 or β-actin (**B** and **C**). Hypothalamic inflammatory cytokine (IL-1β and TNF-α) expression was measured by real-time PCR (**D**). Reported values are relative to GAPDH. Data are represented as mean ± SEM. *P < 0.05; **P < 0.01; ***P < 0.001.

Because we have shown the importance of hypothalamic NF-κB pathway in endotoxemia-induced muscle wasting, we investigated whether the protective effect of knockdown POMC on muscle wasting was related NF-κB. Surprisingly, the NF-κB pathway showed no significant difference between POMC KD and POMC CL rats after LPS administration (Fig. 7A–C), even though they have demonstrated a significant difference in muscle wasting. Besides, hypothalamic inflammation seemed not to be affected either. In the rats injected with saline, there was also no significant difference in hypothalamic signaling pathway and inflammation (Fig. 7D and E). Therefore, the alleviated effect of POMC blockade on muscle was not via NF-κB pathway. On the contrary, combined with the results of experiment 1, NF-κB pathway is more likely to be the upstream of POMC.

### The effect of knockdown POMC on other hypothalamic neuropeptides

**Figure 8:**
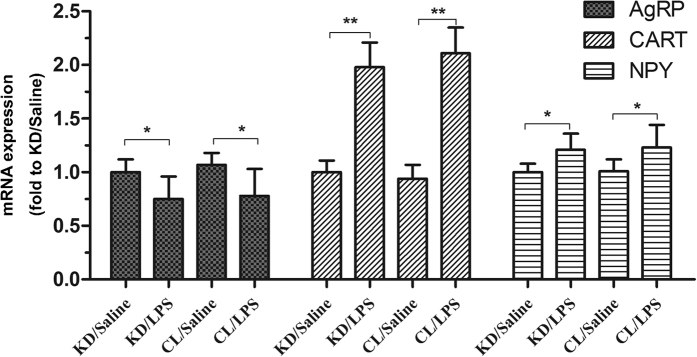
Effects of POMC knockdown on the expression of other hypothalamic neuropeptides. Real-time PCR analysis of hypothalamic AgRP, CART and NPY gene expression was performed in a subset rat of experiment 2. GAPDH was used as control. Data are represented as mean ± SEM. *P < 0.05; **P < 0.01; ***P < 0.001.

Hypothalamic neuropeptides, AgRP, CART and NPY were also determined in the rats of experiment 2. In both POMC KD and POMC CL groups, LPS resulted in decreased AgRP expression, and no difference was observed between the two groups. However, other peptides, CART and NPY, showed an elevation 24 h after LPS injection, without obvious difference between POMC knockdown and control groups either (Fig. 8). These evidences suggested knockdown POMC have little influence on the expression of other neuropeptides in both normal or stressed conditions. The peripheral effect on muscle wasting was more likely related to POMC alone.

## Discussion

In the present study, we firstly demonstrated that inhibiting the activation of central IKKβ/NF-κB pathway could reduce the expression of hypothalamic inflammation and neuropeptides, and more importantly, peripheral skeletal muscle wasting induced by LPS was largely attenuated. Further experiment shown that knocking down the expression of a key neuropeptide, POMC, by a central lentiviral approach can also efficiently ameliorate muscle wasting, without significant effect on central inflammation and other neuropeptides. Taken together, these results indicated that hypothalamic inflammation and neuropeptide played a critical role in acute muscle wasting caused by endotoxemia.

Although the catabolic effects of proinflammatory cytokine on muscle and adipose tissues have been sufficiently studied in peripheral organs^9^, the role of central inflammation in various states is still unclear or even confusing^21^. It was well established that central inflammation played an important, or even decisive role in obesity and type 2 diabetes^10^,^11^. On the other hand, in critical illness or cachexic conditions, emerging data have implied central inflammation may also contributed to the metabolic and behavioral actions^22^,^23^. LPS can elicit significant reduction of feeding in wild-type mice, however, a sustained anorexia cannot be observed in mice with disrupted central inflammatory response, not even after the restoration of peripheral inflammation^24^,^25^. Moreover, central cytokines administration can recapitulate many of the behaviors and features of cachexia^22^. Specifically, rats receiving ICV TNF-α injection presented with reduced body mass and food intake, as well as increased thermogenesis and oxygen consumption^23^. Further inhibition of hypothalamic TNF signaling with ICV infliximab can partially restore the body weight, increase food intake and enhanced survival rate in both septic and tumor-bearing animals. Collectively, these data supported the critical role of central inflammation in cachexic response. Since the key feature of cachexia is muscle wasting^26^, one might postulate that central inflammation may also participate in the process of muscle degradation in critical illness. This was recently studied by Braun and his colleagues^14^. They have elaborately shown central inflammation was necessary and sufficient to induce muscle atrophy. However, the inflammatory pathway within the hypothalamus regulating muscle wasting have not yet been investigated at present. Since peripheral NF-κB was necessary for muscle wasting^9^,^27^, we hypothesized that central NF-κB pathway may participated in the process.

In this experiment, we shown that blockade of central IKKβ/NF-κB pathway with PS-1145 can significantly attenuate hypothalamic inflammation and skeletal muscle wasting caused by LPS. These results indicated activation of central NF-κB pathway was crucial in the cachexic state caused by endotoxemia. And this was in line with other’s researches. Previously, Jang and colleagues have shown NF-κB activation in melanocortin system was essential for the anorexia and weight loss induced by infectious agents and leptin^19^. Similarly, in another model induced by ICV injection of TNF-α, activation of NF-κB was accompanied with a 25% reduction of 12-h food intake as well as increased body temperature and respiratory quotient^28^. Due to the close relationship between NF-κB pathway and inflammation^18^, inhibition of central NF-κB has an impact on the expression of hypothalamic proinflammatory cytokines and neuropeptides as shown in our results, which may further affect the muscle wasting. Although we can see some variation in food intake after NF-κB blockade, this was more likely due to neuropeptides alteration^12^, and should have little impact on peripheral muscle wasting as we have demonstrated in previous researches^29^.

Peripheral LPS injection can lead to a local amplification of central inflammation. These cytokines act upon neural circuits in the hypothalamus to regulate energy balance and metabolism^15^. As known, hypothalamic inflammation has an important role on the melanocortin system, we also found inhibiting NF-κB altered the expression of key neuropeptides, especially POMC and AgRP. Consistently, previous studies have demonstrated NF-κB activation within melanocortin neurons can affect body weight and food intake through modulating different neuropeptides in various conditions^10^,^19^. Thus, we further tested the central and peripheral effect of knockdown key anorexigenic neuropeptide, POMC.

Using a lentiviral method, we successfully inhibited the elevated expression of POMC after LPS treatment. Remarkably, in the knockdown group, LPS-induced skeletal muscle wasting was highly mitigated when compared with the vehicle-treated group, although hypothalamic inflammation or NF-κB pathway was unaffected. This result indicated increased POMC expression was essential for the LPS-induced muscle wasting and hypothalamic inflammation should be the upper stream of POMC. In the study, shRNA treatment did not change the basal POMC mRNA expression significantly, as shown in Fig. 5. We considered this was because the basal POMC mRNA expression is at a very low level, and the method we used is RNA interference, unlike gene knockout, which can inhibit mRNA expression completely.

However, the exact mechanism of POMC mediating muscle wasting remains some speculative. Since POMC could be cleaved into several peptides, including α-MSH, which exert its metabolic effect by binding to MC4R and MC3R^22^, thus the downstream inactivation of melanocortin system may mediate the protective effect of POMC blockade on LPS-induced muscle wasting. Mice with POMC deficiency always resulted in an increase of body lean tissue and fat mass^20^. Besides, they showed higher food intake and lower basal oxygen consumption when compared with the wild type. In addition, disruption of activity at melanocortin receptors, including MC4R and MC3R, also can lead to marked obesity^30^,^31^. On the other hand, in energy wasting diseases, the melanocortin system always showed overactivity^20^, owing to the elevated hypothalamic inflammation. Also, in cardiac cachexia model, mice with a targeted deletion of the MC4R gene gained lean body and fat mass in both sham and myocardial infarction group^32^. In our experiment, inhibition of POMC expression with shRNA can also theoretically prevent the overactivation of MCRs, therefore the alleviated muscle wasting may attributed to this alteration. In our study, downregulation of POMC has little effect on NF-kB and other neuropeptides, which implied these may be the upper stream or independent from POMC. LPS injection can induce profound inflammatory cytokine and other circulating hormones, such as IL-6, leptin and insulin, which all can affect central inflammation and neuropeptides^29^,^33^,^34^.

POMC can also be cleaved into adrenocorticotrophic hormone (ACTH) in the PVN, and activates the hypothalamic-pituitary-adrenal (HPA) axis. And it is well known that increased cortisol is an important inducer of muscle breakdown under the condition of sepsis and cachexia^9^. Researchers have shown cancer- or endotoxin-induced cachexia required intact glucocorticoid signaling in skeletal muscle^14^,^35^. Thus, the protective results obtained in our study may also attributed to the alteration of HPA axis. In our present study, we only measured plasma corticostone, but the results were interesting. In Tables 1 and 2, we can see plasma corticostone other than IL-6 has shown a simiar pattern with change muscle wasting. This result furthur supported the critical role of HPA axis. In addition, Noguerias *et al*. have shown melanocortin outflow to muscle was dependent on the sympathetic innervations of muscle^36^. Also, individuals with functional MC4-R mutation have reduced muscle sympathetic nerve activity^37^. And systemic inhibition of β-3 adrenergic signaling ameliorated the weight loss induced by central inflammation^23^. Therefore, sympathetic nervous pathway may also contribute to the alteration. However, both the two mechanisms require further investigation.

Although, we and others have shown hypothalamic inflammation participated in endotoxemia induced muscle wasting, more data have implicated positive energy balance was relied on central inflammation. This paradox have not yet been fully understood at present. Different inflammatory region, time course, amplitude or neural cells may account for the two opposite process^21^, and this is reviewed elsewhere^11^.

As demonstrated in the present study that hypothalamic inflammation and melanocortin neuropeptide were involved in endotoxemia induced muscle wasting, it is promising that targeting those process may have a better outcome among septic or critical patients. Actually, in clinical practice, only few anti-inflammatory drugs such as celecoxib (COX-2 inhibitor) or etanercept (TNF-blocking agent) have been used in clinical trials for cachexia induced by cancer or rheumatoid arthritis^38^,^39^. Inspiringly, the results were favorable so far. While a few MCR antagonists, such as SHU9119 and ML 00253764, have been used in animal models^20^, there is no published clinical trials. In fact, acute skeletal muscle wasting caused by severe sepsis may not be totally equal to those of chronic illness, thus whether the medicine used in cancer or uremia was still effective among septic patients needs further study. Besides, the new drugs should take the region specificity into account. It should have a high selectivity and can penetrate the blood brain barrier. Maybe, nasal spray can be an effective way for delivering these drugs.

Even though the present study was the first to investigate the role of hypothalamic NF-κB pathway and POMC in acute skeletal muscle wasting caused by infection, it did have some limitations. Firstly, we only used NF-κB inhibitor and shRNA method to block inflammation and neuropeptides, which was inferior to the gene knockout approach, especially the cre-loxp method. Secondly, we only chose 24 h to test muscle wasting due to LPS-injection method, longer time span using other model are warranted to confirm the results. Besides, directly increasing the expression of POMC is important for the research, which will need further study. Also, the exact molecular mechanism underlying POMC regulating muscle wasting required more efforts.

In conclusion, hypothalamic IKKβ/NF-κB pathway activation was essential for acute skeletal muscle wasting induced by endotoxemia. Neuropeptide POMC and melanocortin system may have mediated the contribution of hypothalamic inflammation to peripheral muscle wasting. Therefore, chemicals with the ability of inhibition of NF-κB pathway or melanocortin may have a therapeutic potential for the acute muscle wasting in some infectious patients.

## Materials and Methods

### Animals

Adult male Sprague-Dawley rats weighing 280 ± 20 g were used in this study. Animals were housed in a temperature-controlled room (25 °C) with regular 12:12-h dark-light cycle, and provided with free access to ad libitum and water. All study protocols were approved by the Animal Care and Use Committee at Nanjing University and Jinling Hospital and animals were given at least 7 days to habituate to the environment before the experiments. And all experiments were performed in accordance with the relevant guidelines and regulations.

### Third-ventricle (3V) cannulation

All Rats in experiment 1 underwent surgical implantation of a 26-gauge stainless steel cannula into the third cerebral ventricle (0.7 mm posterior and 8.5 mm ventral to bregma) under pentobarbital sodium (2%, 35 mg/kg) anesthesia, as previously described^40^. After a 7-day recovery period, cannula placement was verified by a positive drinking response after administration of Angiotensin II (40 ng/2 μL), animals that did not drink 5 mL of water within 15 minutes after treatment were not included in the experiment^41^.

### Lentiviruses and Intra-hypothalamic Injections

The lentiviral vector of shRNA against rat POMC and matched control was purchased from GenPharma (GenPharma Co., Ltd Shanghai). The sequences of shRNA was CUCUUCAAGAACGCCAUCA (5′-3′), whose interfering effect was confirmed *in vitro*. Lentiviruses were produced from HEK293T cells through cotransfection of target sequences with their packaging plasmids. Lentiviruses were purified by ultracentrifugation and ~1 × 10^9^ particles/site were used for each virus injection. The bilateral injections to the ARC were directed using an ultra-precise stereotax (Kopf Instruments) to the coordinates of 3.3 mm posterior to the bregma, 9.0 mm below the surface of the skull, and 0.3 mm lateral to midline. Purified lentivirus were injected over 10 min using a 5 μl Hamilton syringe attached to a microinfusion pump (World recision Instruments, Sarasot a, FL). The needle was left for an additional 5 min and then slowly withdrawn.

### Experiment 1: effects of blockade of hypothalamic IKKβ/NF-κB pathway on endotoxemia induced hypothalamic inflammation, neuropeptides expression and muscle wasting

One week after 3v cannulation, rats were divided into four groups according to the following treatment. **Sham**, no treatment; **LPS**, intraperitoneally injection of LPS (10 mg/kg, E. coli, 055:B5, Sigma) only; **LPS/PS1145**, 30 min after i.p. LPS administration rats received an icv injection of PS-1145(10 μg in 10 μl of saline; Sigma), an IKK inhibitor^40^; **LPS/vehicle**, 30 min after i.p. LPS administration rats received an icv injection of vehicle (saline, 10 μl). Twenty-four hours after the treatment, rats were euthanized with an overdose of pentobarbital sodium and the hypothalamus of brain was rapidly dissected and stored at −80 °C until analysis. The rats were weighed at the beginning and the end of the experiment. The choose of LPS dose and time points were according to previous studies, with a mortality around 40–50%^33^,^42^. All the LPS treated subjects showed septic phenomenon, such as shivering, diarrhea or behavioral change. The extensor digitorum longus (EDL) was immediately excised to measure the proteolytic rate, and the Gn muscle was harvested and frozen in −80 °C.

### Experiment 2: effects of knockdown of hypothalamic POMC expression on endotoxemia-induced hypothalamic inflammation, neuropeptides expression and muscle wasting

Two weeks after bilateral injections of interfering or control virus to the ARC, rats were intraperitoneally injected with LPS (10 mg/kg, E. coli, 055:B5, Sigma) or saline. Twenty-four hours after treatment, rats were euthanized and tissues were harvested and stored as described in experiment 1.

### Cardiac Perfusion and hypothalamic Immunostaining

Another set of rats in experiment 2 (5–6 per group) were anaesthetized and transcardially perfused with 200 ml of saline containing heparin (50 i.u./L), followed by 400 ml of 4% paraformaldehyde in 0.1 M phosphate-buffered saline (pH 7.2). Each rat’s brain was removed, post-fixed in 4% paraformaldehyde for 1 h, placed in phosphate-buffered saline containing 30% sucrose and stored at 4 °C. Brain sections of 6 μm thickness were made using a cryostat at −20 °C. Fixed brain sections were blocked with serum of the appropriate species, penetrated with 0.2% TritonX-100, and treated with primary rabbit anti-POMC antibodies, and subsequently reacted with FITC-labeled Goat Anti-Rabbit secondary antibody (Invitrogen). The nucleus was stained by DAPI (4,6-diamidino-2-phenylindole). Images were captured under a FW1000 confocal microscope.

### Tissue Dissection and Western Blot Analyses

Tissue lysis, protein extraction, and Western blot analyses were performed as previously described^27^. Proteins were dissolved in a lysis buffer and separated by SDS/PAGE for Western blot analyses. Primary antibodies included anti-phosphorylated NF-κB (p-p65), anti-NF-κB (p65), anti-β-actin (Cell Signaling Technology, Inc.), and anti-IκBα (Santa Cruz Biotechnology, Inc.). Secondary antibody was HRP-conjugated anti-rabbit IgGs (Pierce). The densitometric analyses of Western blotting images were performed using Image-Pro Plus software (Media Cybernetics).

### Real-time PCR

Total RNA was extracted from hypothalamus using TRIzol reagent according to the manufacturer’s instructions (Invitrogen). Once isolated, 5 μ g of total RNA was reverse transcribed to yield cDNA. Real-time PCR was performed on an ABI Prism 7900 HT (Applied Biosystems, Foster City, CA). Expression levels of each gene were normalized to an internal control gene (GAPDH mRNA) and expressed as % control. The primer sequences used in the study were listed in Table 3.

### Muscle protein breakdown rate

The EDL muscles were used for protein breakdown studies as described in earlier studies^43^. In brief, EDL muscles were tied at resting length to stainless steel supports and incubated for 2 h at 37 °C in Krebs–Henseleit bicarbonate buffer (pH7.4) with 10 mM glucose and 0.5 mM cycloheximide. The total and myofibrillar protein breakdown rates were determined by measuring the net production of tyrosine and 3-methyl-histidine (3-MH), respectively. Levels of both tyrosine and 3-MH in medium or tissue samples were determined by high-performance liquid chromatography (HPLC)^43^,^44^.

### Muscle fiber CSA immunofluorescence

Cryosections of Gn were cut in cross section. For laminin staining, 20μm sections were fixed for 15 min in 4% PFA and then blocked in PBS (10 mM NaPO_4_ and150 mM NaCl)/10% BSA for 1 h at room temperature. Sections were then incubated overnight at room temperature with a rabbit antilaminin antibody (SigmaAldrich) diluted 1:100 in PBS/0.1% BSA, washed with PBS/0.025% Triton X100, and incubated in a goat anti–rabbit Alexa Fluor 555nm labeled secondary antibody (Invitrogen) diluted 1:500 in PBS/10% BSA. Sections were mounted with Vectashield fluorescent mounting media (Vector Laboratories). Images demonstrating fiber type distribution were obtained using a FW1000 confocal microscope. Fiber area was measured in images by Image-Pro Plus software (Media Cybernetics).

### Myocyte ultrastructure examination

The right gastrocnemius was cut into pieces (1 * 1 * 1 mm), fixed with 2.5% glutaraldehyde for 48 h at 4 °C. And they were flushed with phosphate-buffered saline, fixed with 1% perosmic acid, and dehydrated with acetone. Ultrathin sections were placed on 200-mesh copper grids and double stained with 4% uranyl acetate and 0.2% lead citrate. Sections were examined under transmission electron microscopy (TEM, JEM-1010, JEOL, Tokyo, Japan). Ultrastructure of myocyte and mitochondria were observed. The ultra structures were examined by two independent pathologist blinded to the grouping.

### Plasma ELISA and RIA

IL-6 and IGF-1 were measured by ELISA (R&D Systems) according to the manufacturer’s instructions. Plasma corticosterone levels were measured by RIA (MP Biomedicals) according to the manufacturer’s instructions.

### Statistical analysis

Data are expressed as mean ± SEM. Differences were analyzed with one-way ANOVA, followed by student-Newman-Keuls post hoc test for two-group comparisons. A p-value of <0.05 was considered statistically significant. All data were analyzed with SPSS software (version 17.0, Chicago, IL).

## Additional Information

**How to cite this article**: Duan, K. *et al*. Hypothalamic activation is essential for endotoxemia-induced acute muscle wasting. *Sci. Rep.* **6**, 38544; doi: 10.1038/srep38544 (2016).

**Publisher's note:** Springer Nature remains neutral with regard to jurisdictional claims in published maps and institutional affiliations.

## Figures and Tables

**Table 1:** Circulating cytokine and corticosterone in treated rats of experiment 1.

Group	Sham	LPS	LPS/PS114	LPS/vehicle
IL-6 (pg/ml)	21 ± 5^**#**^	234 ± 23*	242 ± 34*	256 ± 40*
Corticosterone (ng/ml)	46 ± 10^**#**^	145 ± 32*	104 ± 39*^#^	165 ± 29*

Plasma IL-6 was measured by ELISA, and corticosterone was measured by RIA. Data are represented as mean ± SEM. *P < 0.05 versus sham. ^#^p < 0.05 versus LPS. Both were calculated by one-way ANOVA.

**Table 2:** Circulating cytokine and corticosterone in treated rats of experiment 2.

Group	KD/Saline	KD/LPS	CL/Saline	CL/LPS
IL-6 (pg/ml)	34 ± 11^**#**^	302 ± 31*	26 ± 7^**#**^	277 ± 54*
Corticosterone (ng/ml)	37 ± 8^**#**^	88 ± 23*^#^	44 ± 12^**#**^	176 ± 49*

Plasma IL-6 was measured by ELISA, and corticosterone was measured by RIA. Data are represented as mean ± SEM. *P < 0.05 versus CL/Saline. ^#^p < 0.05 versus CL/LPS. Both were calculated by one-way ANOVA.

**Table 3:** The primer sequences.

Gene		Primers
MuRF1	Forward	5′-CCAGGTGAAGGAGGAACT-3′
	Reverse	5′-TTGGCACTCAAGAGGAAGG-3′
MAFbx	Forward	5′-CTTGTGCGATGTTACCCA-3′
	Reverse	5′-GTGAAAGTGAGACGGAGC-3′
FOXO1	Forward	5′-CAAGGATAAGGGCGACAG-3′
	Reverse	5′-TTGAGCATCCACCAAGAAC-3′
POMC	Forward	5′-CCTCCTGCTTCAGACCTCCA-3′
	Reverse	5′-GGCTGTTCATCTCCGTTGC-3′
AgRP	Forward	5′-TGAAGGGCATCAGAAGGT-3′
	Reverse	5′-CACAGGTCGCAGCAAGGT-3′
CART	Forward	5′-CCGAGCCCTGGACATCTA-3′
	Reverse	5′-GGAATGCGTTTACTCTTGAGC-3′
NPY	Forward	5′-GTGTTTGGGCATTCTGGCTG-3′
	Reverse	5′-AGTGTCTCAGGGCTGGATCT-3′
IL-1β	Forward	5′-TTCAAATCTCACAGCAGCAT-3′
	Reverse	5′-AGGTCGTCATCATCCCAC-3′
TNF-α	Forward	5′-CCACGCTCTTCTGTCTACTG-3′
	Reverse	5′-GCTACGGGCTTGTCACTC-3′
GAPDH	Forward	5′-GCAAGTTCAACGGCACAG-3′
	Reverse	5′-GCCAGTAGACTCCACGACAT-3′
